# Comparative Analysis of Bariatric Surgery and Non-surgical Therapies: Impact on Obesity-Related Comorbidities

**DOI:** 10.7759/cureus.69653

**Published:** 2024-09-18

**Authors:** FNU Abhishek, Grace D Ogunkoya, Jaikirat Singh Gugnani, Harkamalpreet Kaur, Sakshi Muskawad, Mankaranvir Singh, Gurpreet Singh, Ujjwal Soni, Dhawani Julka, Abasi-Okot Udoyen

**Affiliations:** 1 Internal Medicine, Government Medical College, Amritsar, Amritsar, IND; 2 Family Medicine, Finnih Medical Center, Lagos, NGA; 3 Medicine and Surgery, Anna Medical College, Montagne Blanche, MUS; 4 Medicine and Surgery, Government Medical College, Patiala, Patiala, IND; 5 Medicine, University College of Medical Sciences, New Delhi, IND; 6 General Medicine, National Pirogov Memorial Medical University, Vinnytsia, UKR

**Keywords:** bariatric surgery, gastric bypass surgery, hypertension in the global context, obesity and overweight, obesity treatment, roux-en-y, type 2 diabetes, weight loss intervention

## Abstract

Obesity is associated with a broad spectrum of comorbidities, including metabolic dysregulation, cardiovascular complications, and socioeconomic impacts. Traditional lifestyle interventions often yield transient results in weight management, while bariatric surgery offers a promising alternative. This systematic review adhered to the Preferred Reporting Items for Systematic Reviews and Meta-Analysis (PRISMA) guidelines and focused on randomized controlled trials comparing bariatric surgery (e.g., Roux-en-Y gastric bypass (RYGB), adjustable gastric banding (AGB), and sleeve gastrectomy (SG)) with non-surgical therapies (drug therapy and lifestyle modifications) in the management of obesity-related comorbidities, particularly hypertension and type 2 diabetes mellitus (T2DM). We comprehensively searched databases like PubMed, PMC, and EBSCO using Medical Subject Headings (MeSH) terms related to obesity, bariatric surgery, and non-surgical treatments. We included seven studies involving participants aged 18-64 published within the last six years. We rigorously assessed these studies for quality and extracted data to evaluate outcomes such as weight loss, diabetes remission rates, hypertension management, and renal function. The review found that bariatric surgery consistently resulted in substantial and sustained weight loss compared to non-surgical therapies. Surgical interventions significantly improved hypertension control, reduced cardiovascular risks, and enhanced glycemic control in diabetic patients. The effectiveness of bariatric surgery in promoting diabetes remission was attributed not only to weight loss but also to physiological changes in gastrointestinal anatomy, gut hormones, and energy balance regulation. Limitations identified in the literature included variations in study methodologies, follow-up durations, and patient characteristics, which limited direct comparisons and generalizations. Future research should incorporate more extended follow-up periods and standardized methods to further validate these findings' durability and broad applicability across diverse patient populations. In conclusion, bariatric surgery emerges as an effective treatment option for managing obesity-related comorbidities, particularly hypertension and T2DM. While acknowledging the inherent risks and complexities associated with surgical interventions, ongoing research and clinical innovations are crucial to optimizing patient outcomes and reducing the global burden of obesity-related diseases.

## Introduction and background

The World Health Organization defines obesity in adults as having a body mass index (BMI) equal to or more than 30, whereas a BMI of 25 to 29.9 is classified as overweight [1]. The classification of obesity is shown in Table 1 [2].

**Table 1:** Classification of obesity BMI: body mass index

	BMI (kg/m^2^)	Obesity class
Underweight	<18.5	
Normal†	18.5–24.9	
Overweight	25.0–29.9	
Obesity	30.0–34.9	I
	35.0–39.9	II
Extreme obesity	≥ 40	III

Overweight individuals are affected by a vast array of health issues, such as type 2 diabetes mellitus (T2DM), high blood pressure, heart disease, stroke, sleep apnea, and many more [3]. Hyperglycemia is a hallmark of DM, characterized by impaired insulin action or decreased insulin secretion [4]. Obesity has emerged as a cause of T2DM [5]. Recent research has shown that insulin-dependent decreased glucose absorption in cells is caused by higher plasma levels of free fatty acids [6]. Consistently elevated blood pressure readings define hypertension as the systolic pressure is 130 mm hg or more, the diastolic pressure is 80 mm Hg or above, or both [7]. While age, race, and a positive family history are all considered non-modifiable risk factors for hypertension, the most prevalent modifiable risk variables are smoking, alcohol intake, and obesity [8]. According to research, there are multiple pathways via which obesity leads to hypertension. These include an imbalance in the sympathetic nervous system, increased renin-angiotensin-aldosterone activity, and damage mediated by adipokines [9,10]. Bariatric surgery has become increasingly necessary due to the dramatic increase in the global obesity epidemic over the past 30 years [11]. The current gold standard for treating severe obesity patients is bariatric surgery, which helps patients lose significant weight, lessens the severity of comorbidities, and improves quality of life [12,13]. Surgically altering the esophageal or gastric tube is the main component of bariatric surgery. Indications may involve a high-risk lipid profile, a BMI of 35-40, and a history of DM, high blood pressure, obstructive sleep apnea, or both. Other indications include lack of success with nonsurgical treatment options or a BMI of 30-34.9 in individuals with T2DM who, despite adherence to lifestyle changes and prescribed medication, do not achieve adequate glycemic control [14]. Sleeve gastrectomy (SG), Roux-en-Y gastric bypass (RYGB), adjustable gastric banding (AGB), and biliopancreatic diversion/duodenal switch (BPD/DS) are among the prevalent surgical options [15,16,17]. Despite SG's growth and AGB's decline, research indicates that RYGB remains the preferred technique worldwide [18]. Medications for obesity target neurohormonal dysregulation. Because of hormonal changes brought on by dieting-induced weight reduction, the body is more likely to return to its predefined, higher body weight set point [19]. These changes involve a decrease in leptin and an increase in ghrelin [20]. Currently, nine FDA-approved anti-obesity medications are still available [21], as listed in Table 2.

**Table 2:** FDA-approved drugs for obesity

Name	Year	Mechanism of action / clinical effect
Orlistat	1999	Inhibits intestinal lipase, leading to a reduction in fat absorption of up to 30%
Phentermine-topiramate	2012	Combines a sympathomimetic agent with a carbonic anhydrase inhibitor to suppress appetite and reduce binge eating.
Bupropion-naltrexone	2014	Combination of a dopamine and norepinephrine reuptake inhibitor and a mu-opioid receptor antagonist to reduce appetite and cravings.
Liraglutide	2014	Acts as a GLP-1 receptor agonist to diminish hunger, promote satiety, and increase feelings of fullness.
Gelesis	2019	Utilizes superabsorbent hydrogel particles made from a cellulose-citric acid matrix to promote fullness. Classified as a medical device with therapeutic effects.
Setmelanotide	2020	Functions as a melanocortin-4 receptor agonist to lower appetite.
Semaglutide	2021	GLP-1 receptor agonist that helps to reduce appetite and enhance feelings of fullness and satiety.

This systematic review is designed to analyze and compare the effectiveness, safety, and outcomes of bariatric surgery against non-surgical treatments, such as medications, for managing hypertension, type 2 diabetes, and related health conditions in obese patients.

## Review

Methods

The PRISMA guidelines for conducting systematic reviews were followed [22]. A comprehensive search of the various search engines and databases was done to include relevant literature, including PubMed, PMC, and EBSCO. We explored the databases by using the terms Medical Subject Headings (MeSH), and keywords like "obesity," "bariatric surgery," "non-surgical treatment," "diet therapy," "weight loss," and "drug therapy" were used.

**Figure 1:**
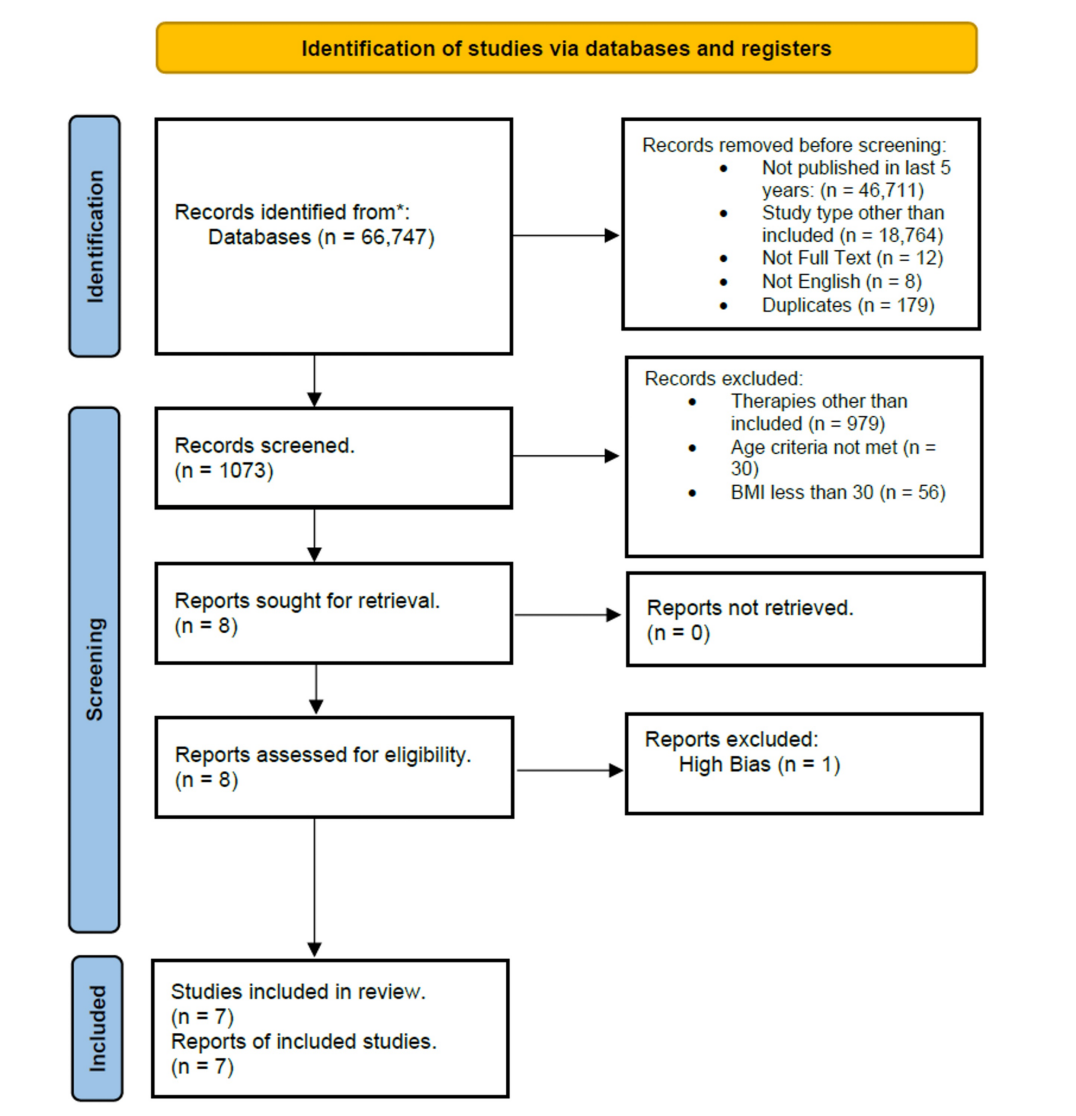
A PRISMA flow chart showing the selection process of included studies

Only randomized controlled trials (RCTs) that compared outcomes between a surgical bariatric group (AGB, biliopancreatic diversion with or without duodenal switch, SG, RYGB) and a non-surgical group (drug therapy and lifestyle modifications) are included in this paper. Eligible articles were required to be full-text, involve participants aged 18-64 years, be written in English, and have been published within the last six years. The primary focus was on comorbidities such as hypertension and DM. Articles were excluded if they were published before 2016, duplicates, or not written in English. In addition, case reports, case series, animal studies, and meta-analyses were excluded. Studies that did not address relevant comorbidities or did not align with the review's objectives were also excluded. A PRISMA flow chart is shown in Figure 1.

The extracted papers underwent rigorous quality assessment using appropriate evaluation tools for each study type. Articles with a minimum non-bias percentage of 50% were considered for inclusion after the assessment. The RCTs that met the criteria are listed in Table 3.

**Table 3:** Quality assessment of the articles.

^Study^	^Non-bias percentage^
[5]	50%
[23]	85.71%
[24]	66.66%
[25]	57.14%
[26]	71.42%
[27]	85.71%
[28]	66.66%

Results

Seven papers were identified and evaluated extensively. Data extracted from the studies are given in Table 4.

**Table 4:** Data compilation from reviewed studies RYGB: roux-en-Y gastric bypass, SG: sleeve gastrectomy, T2DM: type 2 diabetes mellitus, LM: lean mass, FM: fat mass, MWL: medical weight loss, VAT: visceral adipose tissue

Study	Year	No. of participants	Purpose of the study	Follow-up Period	Total weight loss in the surgical group	Outcome	Conclusion
[5]	2020	49	The comparability of obese T2DM patients undergoing RYGB or SG with respect to their diabetes remission rates	Two years	Excess weight reduction was greater following RYGB than following SG; mean ± standard deviation: 78 ± 22 vs. 60 ± 22%, and 76 ± 24 vs. 54 ± 21%.	The study showed that after two years, there was no significant difference in T2DM remission rates between the two operations, despite RYGB showing superior excess weight reduction and a larger percentage of excess weight loss compared to SG at one and two years.	Even though RYGB resulted in greater weight loss, after two years, there was no discernible difference in T2DM remission rates between RYGB and SG. To ascertain SG's function in treating obesity and type 2 diabetes, the study recommends collecting long-term follow-up data.
[23]	2020	61	Remission of T2DM following surgical or nonsurgical treatments.	5 years	-24.9kg	When comparing the surgical group to the people on lifestyle weight loss intervention, the surgical group had the highest percentage of people not needing any medication for T2DM.	For the treatment of type 2 diabetes, surgical procedures are more beneficial than lifestyle changes alone.
[24]	2018	100	Effects on blood pressure of RYGB vs medication alone	3 years	-27.8%	Maintaining blood pressure below 140/90 mmHg while reducing the overall number of antihypertensive drugs by at least 30% was the key goal.	For patients with obesity and hypertension, RYGB is a useful therapy for midterm blood pressure control and hypertension remission, requiring fewer prescription drugs.
[25]	2019	15	To assess the differences in the regional distribution of FM and the relative changes in LM in T2DM subjects who lost weight using MWL, AGB, or RYGB	9 months	NA	AGB lost more LM, RYGB tend to lose more VAT than MWL	At similar weight loss, AGB and RYGB lost more LM and VAT than MWL
[26]	2020	100	The effects of RYGB surgery and medication therapy on albuminuria were studied in patients with obesity, T2DM, and early-stage CKD.	24 months	-25.4%	82% of patients had albumin remission after RYGB when compared with 55% of patients who had only adequate medical therapy.	Remission of albuminuria in patients with T2DM and obesity was more effective in the RYGB group than best medical treatment.
[27]	2018	38	Evaluating the impact of RYGB surgery on obese individuals with type 2 diabetes, clinical and patient-oriented outcomes were measured in comparison to intensive medical diabetes and weight management (IMWM).	3 years	-24.9%	Favorable outcome in cardiometabolic risk for coronary heart disease was noticed in the RYGB group versus IMWM.	Improvement in quality of life, lower HbA1c, reduced cardiovascular risk and weight loss were produced in the surgical group versus IMWM
[28]	2018	88	Effects of metformin and gastric banding on β-cell activity over a two-year period in persons who are moderately obese and have either mild T2DM or Impaired glucose tolerance (IGT).	2 years	-10.7 kg	Both therapies reduced HbA1c levels and improved insulin sensitivity, however the amount of weight loss was different. There was not a significant difference in their effects on β-cell activity or glycemic control.	The study found that during a 2-year period, both gastric banding and metformin had similar effects in maintaining β-cell activity and stabilizing/improving glycemia in this particular cohort.

Discussion

Type 2 Diabetes Mellitus Remission

**Figure 2:**
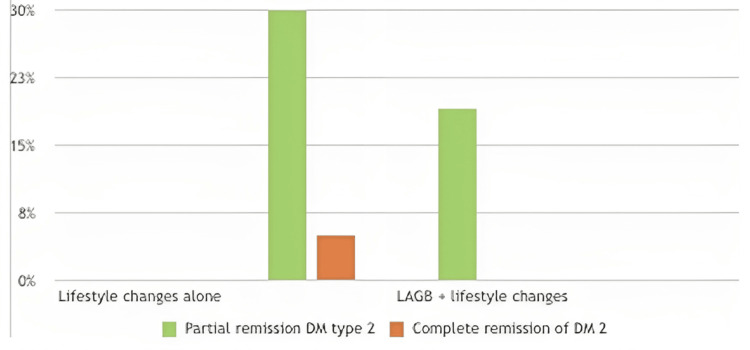
Remission rate of diabetes mellitus (DM) 2

RYGB and laparoscopic adjustable gastric banding (LAGB), when combined with lifestyle changes, are associated with a higher likelihood of T2DM remission compared to lifestyle changes alone [23], as depicted in Figure 2.

Hypertension Management

**Figure 3:**
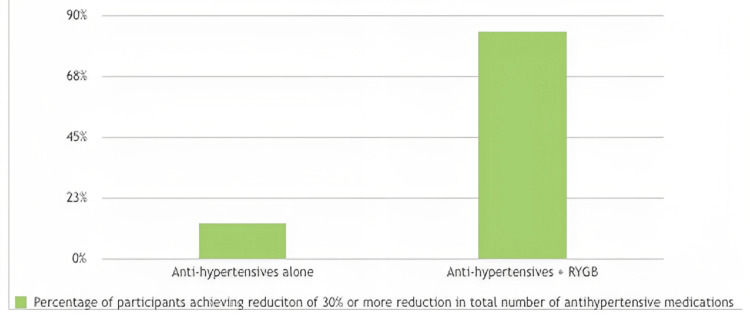
Effect on antihypertensive medication

In the study by Schiavon et al., RYGB is more effective in reducing the total number of antihypertensive medications used to maintain blood pressure. Specifically, patients who underwent RYGB achieved a statistically significant reduction of 30% or more in the total number of antihypertensive drugs, including ACE inhibitors, angiotensin receptor blockers, or calcium channel blockers [24], as depicted in Figure 3.

Renal Function and Chronic Kidney Disease

**Figure 4:**
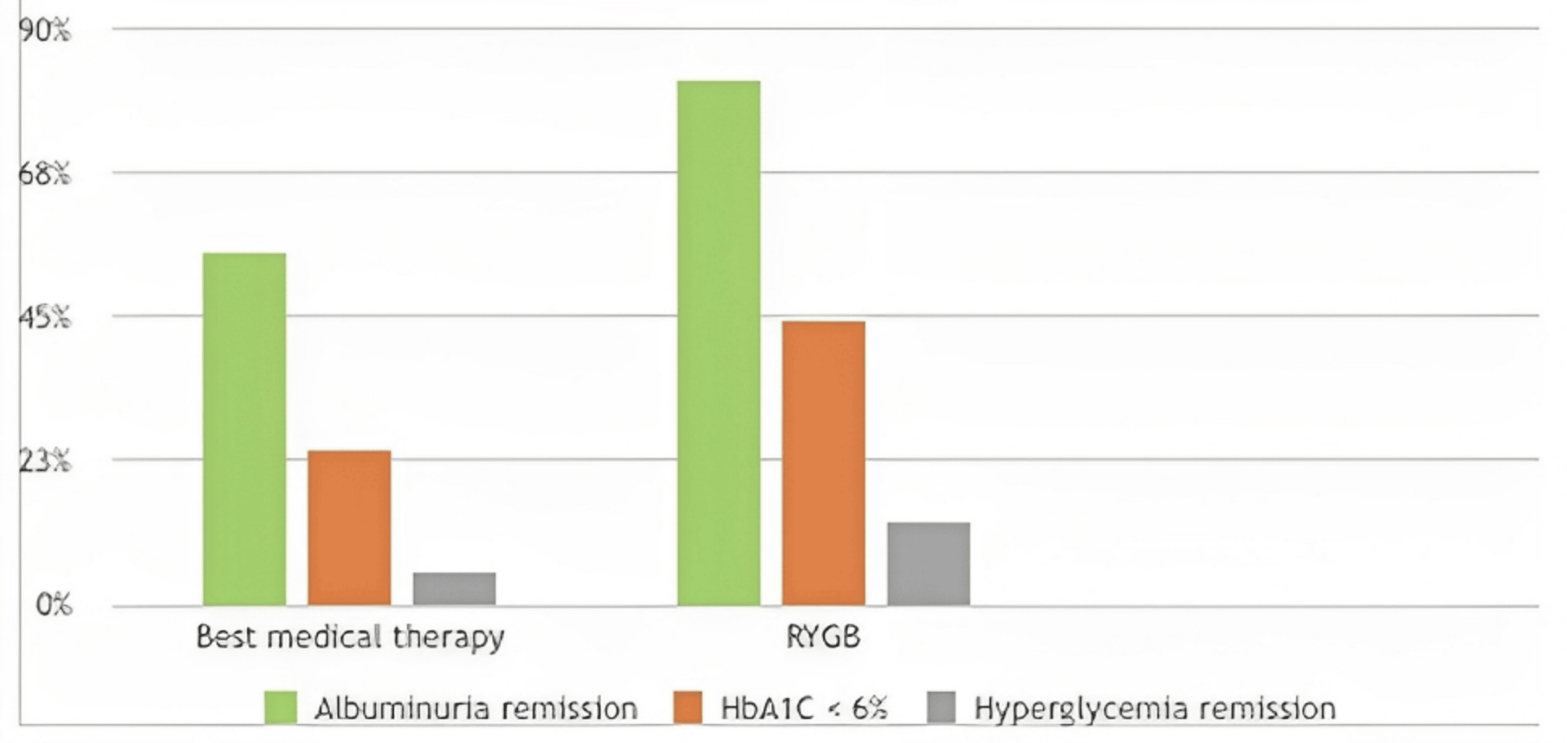
Effect on early stage chronic kidney disease

Patients who have undergone RYGB show improved outcomes in the early stages of chronic kidney disease (CKD) in type 2 DM compared to medical therapy, including drugs like metformin and other medications aimed at optimizing microvascular and vascular outcomes. RYGB is also associated with better control of hyperglycemia [25,26,27], as illustrated in Figure 4.

This systematic review shed light on the diverse impacts of both bariatric surgery and complementary and alternative medicine on a range of critical health indicators. Bariatric surgery emerged as particularly influential, positively affecting glycemic management, weight loss, cardiovascular risk factors, and complications associated with excessive weight. Notably, the review highlighted that bariatric surgery surpasses lifestyle modifications alone in achieving long-term improvements in HbA1c levels and rates of diabetes remission.

The review also underscored that the enhanced diabetes remission rates following bariatric surgery may be attributed to weight loss and physiological changes in gastrointestinal anatomy, gut hormones, and regulatory mechanisms of energy balance. Together, these factors enhance the effectiveness of bariatric surgery as a treatment option.

Limitations

The review acknowledges limitations in the current literature, such as variations in study methodologies, follow-up durations, and patient characteristics, which complicate direct comparisons and generalizations. Future research with more extended follow-up periods and more standardized methods is needed to elucidate further these findings' durability and broader applicability across diverse patient populations.

## Conclusions

Obesity-related comorbidities span from alterations in metabolism and hormones to impacts on cardiovascular health, social well-being, and economic factors. While lifestyle therapies often struggle to achieve and sustain clinically significant weight loss, bariatric surgery emerges as an effective therapeutic option. It consistently leads to substantial weight loss maintained over time, mitigating the fluctuations seen with nonsurgical approaches.

This systematic review aimed to compare and evaluate the benefits and outcomes of surgical versus nonsurgical treatments in randomized controlled trials focusing on hypertension, T2DM, and related comorbidities. The findings unequivocally demonstrate that bariatric surgery, including procedures like RYGB, AGB, SG, and biliopancreatic diversion with or without duodenal switch, effectively reduces a plethora of obesity-related morbidities. These benefits range from improved cardiovascular health, including blood pressure management, to remission of albuminuria in patients with chronic kidney disease. Bariatric surgery also significantly enhances glycemic control, maintaining HbA1C levels within optimal ranges for diabetic patients, thereby improving overall quality of life.

Long-term success with both bariatric surgery and nonsurgical therapies hinges on holistic and interdisciplinary approaches. Adopting healthier habits such as improved nutrition, regular exercise, and behavioral modifications is central to achieving sustained weight control and favorable outcomes in both treatment modalities. Continuous medical and psychological support is essential to overcoming challenges and improving long-term health.

While bariatric surgery offers substantial benefits in weight loss and metabolic health, it has risks, such as infection, hemorrhage, and long-term vitamin and mineral deficiencies. Rigorous post-operative monitoring and adherence to prescribed supplements and dietary regimens are crucial to mitigating these risks and optimizing health outcomes.

By contrast, nonsurgical therapies are less invasive and generally safer but may not achieve the same magnitude of weight loss as surgical interventions. Nonetheless, they are valuable in improving overall health and reducing obesity-related complications when adhered to consistently and diligently.

Physicians should tailor the choice between bariatric surgery and nonsurgical therapies to each patient's specific circumstances, including their level of obesity, presence of comorbidities, lifestyle preferences, and individual goals. A collaborative decision-making process involving healthcare providers, patients, and their support networks is essential to developing personalized treatment plans that optimize outcomes.

In conclusion, both bariatric surgery and nonsurgical therapies for obesity offer distinct benefits and considerations. Bariatric surgery provides significant and enduring weight loss compared to nonsurgical approaches focused on behavioral modifications, thereby substantially improving health outcomes. The success of either approach depends on sustained commitment and support. Continued advancements and research in bariatric surgery are warranted to alleviate the physiological, economic, and emotional burdens of obesity, empowering healthcare providers to serve their patients better and improve public health.
